# Fabrication of a Soft Robotic Gripper With Integrated Strain Sensing Elements Using Multi-Material Additive Manufacturing

**DOI:** 10.3389/frobt.2021.615991

**Published:** 2021-11-01

**Authors:** Antonia Georgopoulou, Bram Vanderborght, Frank Clemens

**Affiliations:** ^1^ Department of Functional Materials, Empa–Swiss Federal Laboratories for Materials Science and Technology, Dübendorf, Switzerland; ^2^ Department of Mechanical Engineering (MECH), Vrije Universiteit Brussel (VUB), and Flanders Make, Brussels, Belgium

**Keywords:** fused deposition modeling, soft robotics, strain sensor, piezoresistive sensor, additive manufacturing

## Abstract

With the purpose of making soft robotic structures with embedded sensors, additive manufacturing techniques like fused deposition modeling (FDM) are popular. Thermoplastic polyurethane (TPU) filaments, with and without conductive fillers, are now commercially available. However, conventional FDM still has some limitations because of the marginal compatibility with soft materials. Material selection criteria for the available material options for FDM have not been established. In this study, an open-source soft robotic gripper design has been used to evaluate the FDM printing of TPU structures with integrated strain sensing elements in order to provide some guidelines for the material selection when an elastomer and a soft piezoresistive sensor are combined. Such soft grippers, with integrated strain sensing elements, were successfully printed using a multi-material FDM 3D printer. Characterization of the integrated piezoresistive sensor function, using dynamic tensile testing, revealed that the sensors exhibited good linearity up to 30% strain, which was sufficient for the deformation range of the selected gripper structure. Grippers produced using four different TPU materials were used to investigate the effect of the Shore hardness of the TPU on the piezoresistive sensor properties. The results indicated that the *in situ* printed strain sensing elements on the soft gripper were able to detect the deformation of the structure when the tentacles of the gripper were open or closed. The sensor signal could differentiate between the picking of small or big objects and when an obstacle prevented the tentacles from opening. Interestingly, the sensors embedded in the tentacles exhibited good reproducibility and linearity, and the sensitivity of the sensor response changed with the Shore hardness of the gripper. Correlation between TPU Shore hardness, used for the gripper body and sensitivity of the integrated *in situ* strain sensing elements, showed that material selection affects the sensor signal significantly.

## Introduction

Fused deposition modeling (FDM) is a simple, low-cost method of additive manufacturing (AM) for the fabrication of 3D objects made from thermoplastic materials (Hwang et al., 2015). It is a widespread method that involves the extrusion of thermoplastic polymers through a heated hot-end and deposits the extruded polymer thread layer by layer on a moving platform to build up a 3D structure (Ligon et al., 2017). Besides, thermoplastic elastomers like polyurethanes can be used for FDM technology (Barnett and Gosselin, 2015; Georgopoulou and Clemens, 2020). TPU filaments for FDM-based printers are commercially available nowadays, and they can reach elongations of up to 700% (Tadesse et al., 2017; Haryńska et al., 2018). TPU filaments used for FDM are interesting for various applications where large elongations are required, like stretchable electronics and soft robotics (Lee et al., 2017; Roels et al., 2020). Commercial filaments like NinjaFlex show good printability with high resolution for applications that involve superior durability (Farstad, 2017; Nelson et al., 2019). Ramadan and Dahle (2019) used commercial NinjaFlex filaments to print complex elastic geometries. However, there has not been a systematic study to address how material selection should be performed for soft robots produced by FDM 3D printing.

Combining TPU with carbon black fillers leads to conductive filaments that are compatible with desktop-size 3D printers (Gnanasekaran et al., 2017). Manganiello et al. (2019) explored the printing of commercial TPU using conductive fillers for manufacturing of force-sensing devices. A commercial conductive TPU-based filament for strain sensing has been investigated by Al-Rubaiai et al. (2019). However, the working range between 0 and 12.5% strains is a limiting factor for many applications. Christ et al. developed conductive filaments based on TPU and CNTs. The printed structures had excellent sensitivity (gauge factor GF = 176), with an elongation up to 100% (Christ et al., 2017). Nevertheless, the dynamic response of the sensor showed nonlinearity in the unloading, and the relaxation behavior of the sensor was not explored.

Multi-material processing methods like FDM printing allow fabrication of structures with the combination of two or more materials and are particularly interesting in the case of embedding conductive materials during printing (Emon, 2019). The combination of different materials contributes to the merging of different desired properties that come from each material phase in the resulting structure (Mansouri et al., 2018). Especially in the field of soft robotics, a combination of excellent flexibility of the printed body and the *in situ* embedding of strain sensing elements is of high interest, and therefore, multi-material 3D printing is required (Shih et al., 2019). Combining printing of conductive and nonconductive filaments leads to the fabrication of complex robotic structures, like actuators with integrated sensing capabilities (Micalizzi et al., 2019). However, there are limited studies that explore the potential of multi-material FDM for soft robotic applications. The entry of commercial conductive and nonconductive filaments into the market provides a lot of possibilities for creating 3D-printed embedded sensors embedded in soft robots. The commercial filament FilaFlex was used for the fabrication of a prosthetic finger that was used as a substrate for sensor fabrication (Sencadas et al., 2017). The study showed the potential of using commercial filaments to integrate sensor properties inside robotic applications; however, it would be an advantage to print the sensor and the soft robotic structure *in situ* and in a one-step process.

Based on this background, we investigated the *in situ* printing of strain sensing elements in soft robotic structures, like soft grippers, using multi-material FDM. Based on our previous studies on the effect of different elastomers on the strain sensing behavior of the soft piezoresistive fibers (Georgopoulou et al., 2020a), we studied the effect of the TPU Shore hardness, used for the gripper structure, on the sensor performance of *in situ* printed TPU-based piezoresistive elements. *In situ* printing of strain sensing elements using multi-material AM with a commercial FDM 3D printer was shown in a previous study (Georgopoulou et al., 2021b). However, in that case dynamic, cyclic tests were not performed, and only one strain level was investigated. Flandin et al. (2001), Culha et al. (2014), and Georgopoulou et al. (2020c) have shown that integration of fiber-based piezoresistive soft sensors in elastomer structures has a good potential to measure the electrical resistance change during extension of the structure. Therefore, properties like the electromechanical drift and relaxation of the *in situ* printed multi-material structures were investigated in the current study, at different strain levels, to explore the optimal strain region for future 3D-printed soft strain sensing TPU structures. These important parameters of the sensor behavior were defined by tensile testing. It is important to address how the material selection and combination between the substrate and the sensor material can have an impact on the sensor response. This aspect can guide future soft robotic fabrication approaches. The sensor response was then also investigated when integrated in the soft robotic gripper, showing the ability of the sensor for obstacle recognition and monitoring the position of the gripper.

## Materials and Methods

### Flat TPU Substrates With Integrated Strain Sensing Elements

For the study, commercially available thermoplastic elastomer-based filaments with various Shore hardnesses were obtained from different companies: FilaFlex 70A, FilaFlex 82A, and FilaFlex 95A (Recreus Industries, Elda, Spain), NinjaFlex (Fenner Drives, Manheim, United States), FiberFlex 40D from Fiberology (Fiberlab, Brezie, Poland), and Yousu 98A from Guangzhou YouSu (Yousu, Guangzhou, China). Nowadays, only one conductive TPU-based filament is available on the market, the Eel (Fenner Drives, Manheim, United States). For the FDM printing of the soft robotic gripper with integrated strain sensing elements, a commercial multi-material printer, BCN3D Sigma R19 3D (BCN3D Technologies, Castelldefels, Spain), was used. After some optimization steps, all the materials could be printed with the same printing parameters. A temperature range between 220°C and 235°C was attempted for the nozzle temperature. Below 230°C, the flow from the nozzle was inadequate, causing under-extrusion. Above 230°C, there was over-extrusion and excessive stringing. The temperature for the printing bed was selected based on the adhesion of the structure on the printing bed. Temperatures below 45°C lead to insufficient adhesion. To avoid internal stresses, the higher temperature was not investigated. The value for the layer height was considered a compromise between the total printing time and the quality of the resulting fabricated object. An infill angle of 90° was used. For the extrusion multiplier, a value of 1.7 was used to achieve sufficient extrusion and flow of the melted material. The value is considered high in comparison to other thermoplastics; however, it is worthwhile to mention that this material has significantly higher elastic properties. No retraction was used during the printing. The first layer was printed with the same speed as the other layers. A higher printing speed than 15 mm/s leads to a bad quality of the printed part because of the under-extrusion problem.

In order to evaluate the different material combinations, a simple rectangular design with an integrated *in situ* strain sensing element was investigated using tensile testing (Supplementary Figure S1). TPU substrates with the dimensions 130 × 10 × 0.3 mm were printed using one nozzle. The selected layer height was 0.2 mm, but the thickness of the strip after the printing was measured to be 0.3 mm because of the elastic properties of the TPU. This difference was attributed to the die swell effect. Finally, the strain sensor element with the dimensions 130 × 0.6 × 0.3 mm was printed on the surface of the TPU using a second nozzle. We later call these printed elements TPU strips.

The sensor strip consisted of the substrate with the sensor line printed on the top in the middle of the substrate. This structure was inspired by the previous work based on fiber composites (Georgopoulou et al., 2020b; Georgopoulou et al., 2021a). A fiber composite has the significant advantage that it results in a sensorized structure without significantly increasing the stiffness of the structure. Furthermore, this structure was considered to be compatible with the fabrication method. Since fused deposition modeling contains the layer-by-layer deposition of the thermoplastic material into building a 3D model, having the sensor fiber on the top seemed a suitable approach.

### Measuring Shore Hardness

Since the Shore hardness was the parameter that was different between the different TPU materials, the Shore hardness of the different filaments was assessed. The Shore hardness was measured using an HBA handheld analog durometer (Sauter, Balingen, Germany). According to DIN 53505, the 3D-printed TPU substrates were folded into 6-mm-thick pieces to measure the Shore hardness using the durometer.

### Tensile Testing

For the tensile testing, a Zwick Roell Z500 (ZwickRoell, Ulm, Germany) with a 200N X force P load cell was used. The samples were fixed onto the tensile machine using pneumatic grippers (4 bar). The use of the pneumatic clamps ensured the avoidance of slipping during the tensile testing experiments. To assess *in situ* the piezoresistive response of the integrated strain sensing elements, a Keithley 2450 source meter (Keithley Instruments, Solon, United States) was used. KickStart software from Keithley Instruments was used in combination with the source meter to monitor the electrical resistance. Because of the high ohmic behavior of the printed piezoresistive sensor part, a two-terminal sensing mode was used to investigate the resistance by measuring the current, using a constant voltage of 1 V. In order to calculate the deviation in the value of the resistance, the standard deviation was calculated between five different samples. The coefficient of the variation was defined by dividing the value of the deviation with the initial resistance (*R*_*0*_). A strain rate of 200 mm/min was used for the tensile tests. Three types of tensile tests were performed to evaluate the performance of the different *in situ* printed combinations for the conductive and nonconductive TPUs. A tensile test up to the point of fracture was used as a first evaluation for the sensing behavior. The Young’s modulus was evaluated based on the DIN 53504. Therefore, the Young’s modulus was calculated to be the slope of the stress–strain curve in the elastic region. The elastic region was defined as the constant stress–strains slope below 1% deformation.

In a second approach, the dynamic behavior was evaluated using the cycling tensile test. The dynamic tensile test was performed, with ten repeatable cycles of straining and releasing. First, the printed TPU strips with the integrated sensing elements were tested to evaluate the dynamic performance at low strains of 0–30%. Later, additional dynamic tests were performed from 0 to 100% and between 90 and 100%. Based on the results of the dynamic testing, the signal drift was calculated. The signal drift was defined as the percentage difference of the relative resistance (Supplementary Figure S2), at the same strain between cycles 2 and 10. The strain where a secondary peak appeared during the dynamic testing was defined as uncertainty (Supplementary Figure S2A). A quasi-static test was used to analyze the relaxation behavior of the printed TPU strips. Therefore, a cycling tensile test at strains between 0 and 30% with a dwell time of 30 s at the maximum and minimum strain levels was performed. The mechanical relaxation was defined as the percentage difference of the value of the stress, at the beginning and end of the dwell time. The electrical relaxation was defined as the percentage difference of the value of relative resistance, at the beginning and end of the dwell time.

Because of the variation of the initial electrical conductivity, the relative resistance was calculated using Eq. 1, where R is the measured resistance and $R_{0}$ is the value of the resistance at the beginning of the measurements.

$$R_{rel}=\frac{R-R_{0}}{R_{0}}$$ (1)

### Soft Robotic Gripper Based and TPU Material With Integrated Strain Sensing Elements

**FIGURE 1:**
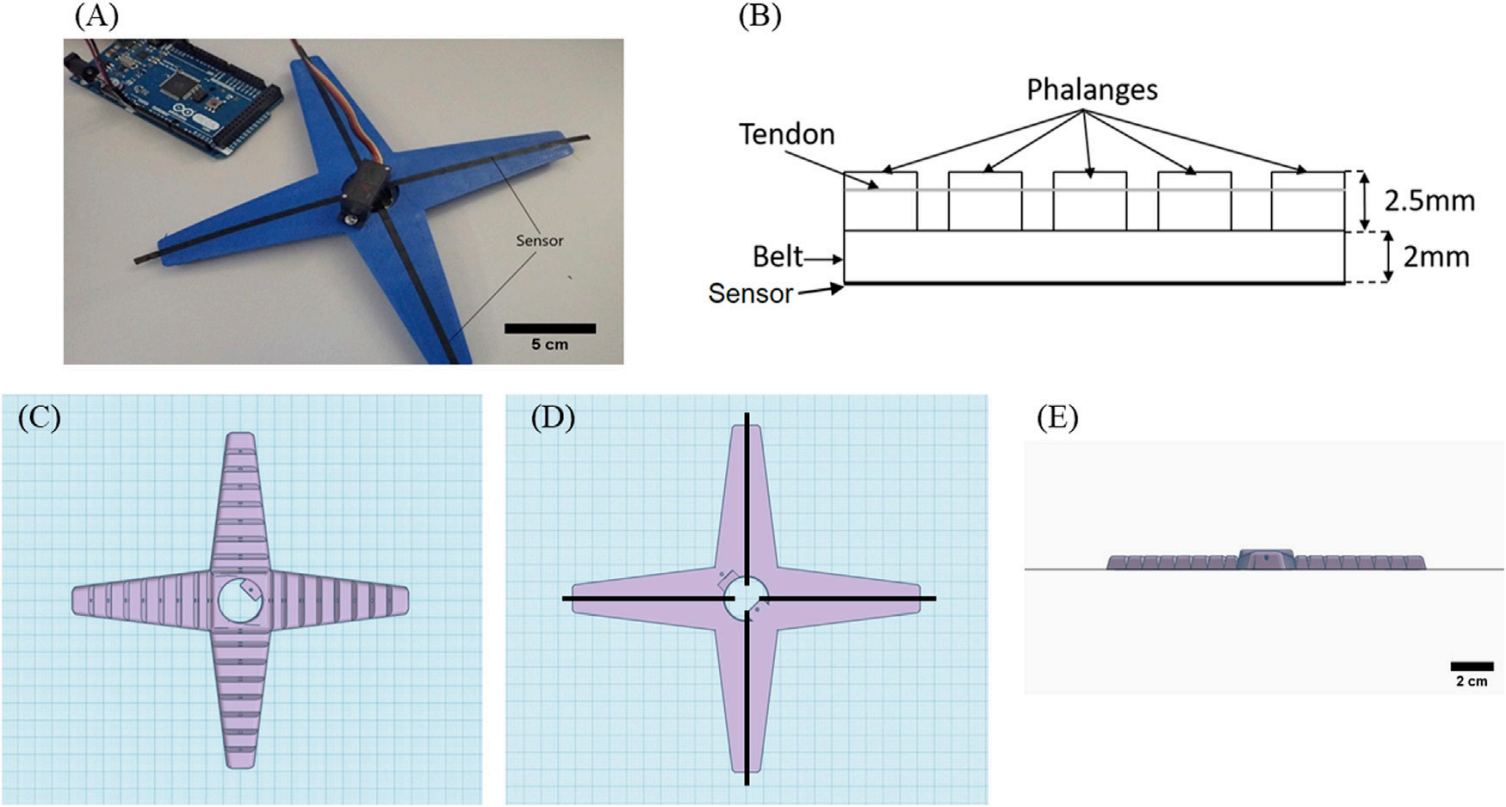
**(A)** Soft robotic gripper with integrated strain sensor elements produced with multi-material FDM printing. **(B)** Sketch of a tentacle, showing the belt, the phalanges, and the wires (tendons) which are connected to the servomotor and the CAD design of the gripper. **(C)** Top view. **(D)** Bottom view. **(E)** Side view. This is an open-source design (Print-in-Place Robotic Gripper).

Similar to the printed TPU strips, the open-source soft robotic gripper structure (Figures 1C–E) was manufactured using a BCN3D Sigma R19 3D with two printing heads, too. For the printing of the soft grippers, the parameters reported for the TPU strips were used. Because of the design (Figure 1B), first, the sensor element was printed with the conductive Eel filament. The size of the sensor was 100 × 0.6 × 0.3 mm. The width and thickness of the sensor were the same as the ones used for the strips. Only the length had to be adapted to match the dimensions of the tentacle of the gripper. Later, the gripper structure was printed with the nonconductive TPU filaments (Figure 1A).

The sensing elements were printed across the main axis of each tentacle of the gripper. An adequate quantity of glue, Magigoo Pro Flex (Thought3D Ltd., Paola, Malta), was necessary to ensure sufficient adherence to the printing bed. This glue was only needed for the larger structure of the gripper and not for the structure of the strips with the integrated sensing elements. Warping occurred above the 30th layer, linked with the thermal shrinkage of the thermoplastic elastomer. Thermal shrinkage occurred because of the high thermal expansion coefficient that resulted in internal stresses. Above the 30th layer, the internal stress became so high that warping occurred. Increasing the temperature of the printing bed was not successful in addressing the problem, and therefore, the use of glue was deemed necessary. After the printing of the gripper, stranded wires, 0.1 mm in diameter, used for electronic applications, coated with silver, were inserted at each of the four tentacles to act as tendons. A change in the length of the tendon of 30% was measured when the tentacles of the grippers moved from the open to the closed position. These tendons were necessary for the motion of the soft robotic gripper. The tendons were made of metal wires that were winded and rolled up using a Tower Pro MG90S micro servo (Adafruit Industries, New York, United States). The control of the servo motor was performed using an Arduino microcontroller. The piezoresistive sensing elements were connected to the Keithley 2450 source meter. Moving the tentacles by the servo motor, the electrical signal of the piezoelectric sensor was measured.

The suggested application for the gripper in this study is the packaging of fruit. The flexibility of the soft gripper makes it a good option for the handling of sensitive objects, like foodstuff and fruit. With that purpose in mind, two different objectives were chosen: a 90-g clementine orange (big object) and a 12-g strawberry (small object). The two objects were placed in the middle of the gripper to be gripped by all the tentacles at the same time. The signal was recorded when the gripper was gripping the big object, then moved to position open, then the small object, and then moved to position open again.

### Optical Microscope Imaging

The cross-section of the strips was investigated using a light microscope (Carl Zeiss AG, Jena, Germany). To avoid smearing of the TPU support and TPU sensor material into each other, the samples were immersed in liquid nitrogen and then cut, so that the cross-section could be analyzed using a software tool (Imagic, Imagic Bildverarbeitung AG, Opfikon, Switzerland).

## Results

### Analysis of the 3D-Printed Samples

All materials were printed with the same parameters, and the real dimensions were investigated by caliper, micrometer, and optical microscopy. From the measurements (Supplementary Table S1), it can be observed that there is no significant change in the length (130 mm) for the substrates printed with different TPUs. For the width, it can be seen that for the substrates printed with FilaFlex 82A, NinjaFlex 85A, and FiberFlex 40D, the values are between 10.1 and 10.3 mm, while for FilaFlex 95A and Yousu 98A, the values are slightly higher (10.6 mm). A similar observation can be seen for the thickness of the printed substrates. For the FilaFlex 82A and NinjaFlex 85A, a thickness of 0.31 and 0.32 mm could be achieved, respectively. For the FiberFlex 40D, the value increased slightly to 0.38 mm, whereas for the FilaFlex 95A and Yousu 98A, even a thickness of 0.47 and 0.535 mm could be measured, respectively. Due to the reason that all substrates were printed with the same parameters, it can be assumed that the different die swelling of the different TPUs will cause different dimensions in thickness and width (Nikzad et al., 2011; Shadvar et al., 2021). It is obvious that those strips that show higher width also result in higher thickness because of the die swell effect. In Supplementary Figure S3, the cross-section of the printed strips including the sensor element is shown. As mentioned, all samples were printed with a constant layer height of 0.2 mm. For the two strips based on FilaFlex 95A and Yousu 98A, a part of the conductive TPU material smeared around the tip of the printing nozzle. This can be explained by the smaller gap caused by the higher swelling of those two TPU materials and will result in a significantly lower thickness of the printed sensor element (smaller volume of the deposited sensing element on the substrate), as shown in Supplementary Figure S3.

### Tensile Test up to the Point of Fracture

**FIGURE 2:**
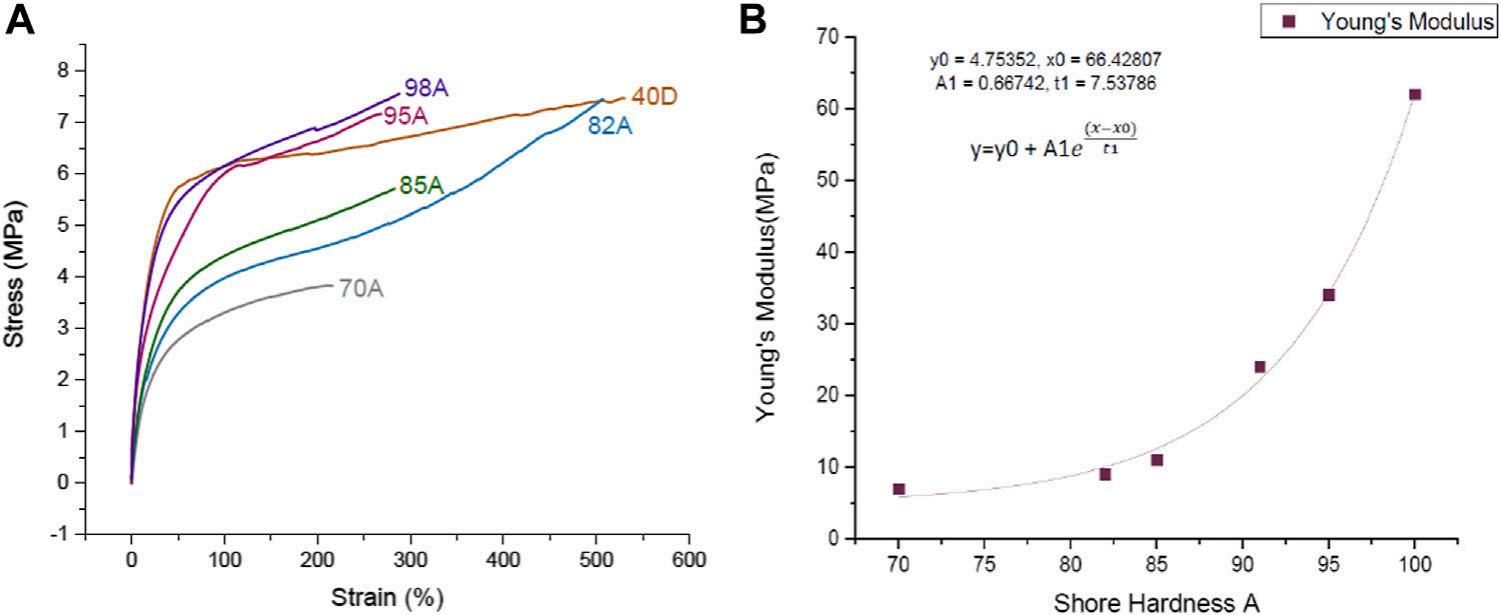
**(A)** Stress–strain curve up to the point of fracture for the 3D-printed TPU strips with *in situ* integrated sensing elements using TPU filaments with different Shore hardnesses. (FilaFlex 70A, 82A, 95A, and 40D; NinjaFlex 85A; Yousu 98A). **(B)** Correlation of the Young’s modulus and the Shore hardness of the 3D-printed TPU strips without the strain sensing element. The plot was fitted as a one-phase exponential growth with time offset.

Multi-material FDM printing, with the conductive Eel filament and the different nonconductive commercial TPU filaments, was done to evaluate the sensing properties. Flat printed substrates with strain sensing elements were investigated by tensile testing. From the stress–strain curve (Figure 2A), it can be seen that the Shore hardness of the substrates affected the strain at the point of fracture. In general, a higher Shore hardness resulted in a higher strain at the point of fracture and ultimate strength.

It has been seen in previous attempts that the properties of the system can be altered after the printing (Christ et al., 2017), and for that reason, the Shore hardness of the samples was measured after the printing (Table 1). By comparing the Shore hardness given by the supplier of the TPU filaments and the printed TPU structure, it can be observed that the Shore hardness slightly decreased after the printing. Based on the DIN 53504, the Young’s modulus was calculated for the different 3D printed strips. Figure 2B shows the relationship between Shore hardness and the Young’s modulus of the printed TPU strips. A good correlation between Shore hardness and Young’s modulus can be observed.

**TABLE 1:** Mechanical properties of the printed TPU filament without integrated *in situ* sensing elements. The Young’s modulus and elongation at the point of fracture are derived from Figure 2.

Filament	Shore hardness given by the supplier	Shore hardness after printing (A)	Young’s modulus substrate (MPa)	Elongation at point of fracture (%)
FilaFlex 70A	70A	68	7	215
FilaFlex 82A	82A	78	9	506
NinjaFlex 85A	85A	81	11	282
FiberFlex 40D (∼90A)	40D	87	24	529
FilaFlex 95A	95A	91	34	262
Yousu 98A	98A	94	62	287

**FIGURE 3:**
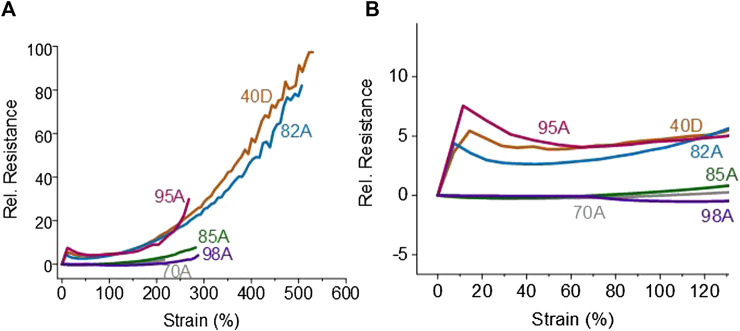
Relative resistance–strain curve for the 3D-printed TPU strips with *in situ* integrated sensing elements using TPU filaments with different Shore hardnesses. (FilaFlex 70A, 82A, 95A, and 40D; NinjaFelx 85A; Yousu 98A) **(A)** up to the point of fracture and **(B)** close-up at strains up to 130%.

In addition to the stress–strain analysis, the electrical response up to the point of fracture was investigated (Figure 3A). The printed structures do not show a constant slope up to the point of fracture. For the strips made with FilaFlex 82A, FilaFlex 95A, and FiberFlex 40D filaments, an increase in relative resistance below 20% strain can be seen. After this first increase, the relative resistance slightly decreased to a minimum. Later on, the relative resistance slightly increased up to 120% (see Figure 3B). With further straining up to the point of fracture, the relative resistance increased significantly.

For the strips printed with the other three TPU filaments, the relative resistance does not change significantly up to a strain of 80%. It could be assumed, even though detachment between the sensor and the substrate was not observed, that the interaction between the integrated sensing element and the TPU substrate was not perfect, and therefore, the strain transfer between the substrate and the sensing element was not sufficient. The FilaFlex 95A, the FiberFlex 40D, and the FilaFlex 82A had a higher sensitivity, expressed by the slope of the relative resistance/strain plot, than the FilaFlex 70A, the Yousu 98A, and the NinjaFlex 85A.

However, at higher strain values (higher than 350%), a significant noise of the electrical resistance (sensor signal) appeared. It can be expected that the noise of the electric signal belonged to slipping effects at a very high elongation of the printed strips inside the pneumatic grippers of the tensile machine.

### Dynamic Tensile Testing

Even though a tensile test up to the point of fracture is a good method to predict the sensitivity of the integrated sensing elements, dynamic tensile testing is proposed to evaluate the sensor signal behavior under cycling conditions (Georgopoulou et al., 2020a). Based on some calculations on the open-source–designed soft gripper structure used for this study, a maximum strain of 30% can be expected for the *in situ* integrated sensing elements.

It was observed during the dynamic testing that stress became zero or negative while releasing the strain for some printed strips (Supplementary Figure S4). The negative stress can be explained by the buckling during the cycling test. Buckling is a well-known phenomenon in tensile testing of elastomers because of the viscoelastic behavior of the elastomer (e.g., creep). Optical observation during the dynamic tensile test confirmed the appearance of buckling at low strains. The presence of buckling confirmed the presence of viscoelastic effects, especially at areas of low strains, where the buckling appeared. The strain values, at which the buckling occurred, are summarized in Table 2. Buckling occurred at around 8% strain, except from the NinjaFlex 85A, where buckling could be observed at 3% strain. Looking at the values of the initial resistance *R*_*0*_, an increase in Shore hardness of the TPU strips resulted in an increase in the *R*_*0*_. Except for the strips based on the FiberFlex 40D filament, the other strips fit into this trend (see Table 2). The differences in the *R*_*0*_ can be linked with the printing process. Even though the sensor material was the same for all the strips, the printing on a different substrate resulted in differences in the *R*_*0*_. Printing the samples on a substrate of higher Shore hardness leads to not only a higher value in the *R*_*0*_ but also a larger variation. The printing of the sensor line on substrates of lower Shore hardness can lead to better consistency in the sample quality. As for the cross-section of the samples, it was seen that the area of the sensing elements decreased by increasing the Shore hardness of the TPU substrate. This observation was expected, thanks to the different thicknesses of the substrate strips that were attributed to the die swell effect discussed previously (Supplementary Table S1). A smaller cross-section can explain the opposite trend for the *R*_*0*_, as mentioned above. However, similar to the initial resistance, the FiberFlex 40D does not fit in the trend. Looking on the resistivity, it can be observed, however, that the resistivity did not follow this trend. Therefore we assume that the different TPU materials used for the substrates will have an effect on the initial resistance of the strain sensor element. As shown in Supplementary Figure S3, a difference observed in the morphology of the strips can affect the differences in the *R*_*0*_ for the FilaFlex 95A and Yousu 98A. The cross-section of the printed elements was more round for the lower Shore hardness and more elongated (with a higher width) for the higher Shore hardness. Such differences can be associated with the printing process. As mentioned in *Flat TPU Substrates with Integrated Strain Sensing Elements*, the substrate is printed first and the sensing element on the top of it. From the results, it can be assumed that the sensors printed on a lower Shore hardness resulted in a different morphology in comparison to the two with high Shore hardness.

**TABLE 2:** Values of the initial resistance *R*_*0*_, the coefficient of variation, and the strain values where buckling and uncertainty can be overserved as well as the signal drift at 30% strain and the hysteresis at 15% strain for the different TPU-based strips during the dynamic tensile test at strain 0–30%. The buckling is derived from Supplementary Figure S4. The cross-section of the samples was calculated based on images using the optical microscope in Supplementary Figure S3. The uncertainty, drift, and hysteresis are derived from Figure 4.

Substrate material	*R*_*0*_ ^a^ (kΩ)	A^b^ (mm^2^)	ρ^c^ (Ωm)	CV^d^ (%)	BS^e^ (%)	US^f^ (%)	SD^g^ (%)	HS^h^ (%)
FilaFlex 70A	48 ± 3	0.76	0.29 ± 0.02	6	8	6	37	66
FilaFlex 82A	54 ± 2	0.72	0.30 ± 0.01	4	8	3	44	23
NinjaFlex 85A	56 ± 1	0.70	0.30 ± 0.01	2	3	0.5	10	46
FiberFlex 40D (∼90A)	43 ± 2	0.73	0.23 ± 0.01	5	8	1	42	17
FilaFlex 95A	177 ± 23	0.50	0.69 ± 0.09	13	8	1	42	31
Yousu 98A	342 ± 40	0.34	0.9 ± 0.1	12	9	6	30	61

a R_0_, Initial resistance.

b A, Cross-section of the printed strain sensor element.

c ρ, Resistivity.

d CV, Coefficient of variation.

e BS, Buckling at strain.

f US, Uncertainty at strain.

g SD, Signal drift.

h HS, Hysteresis of the signal during the 5th cycle at 15% strain.

**FIGURE 4:**
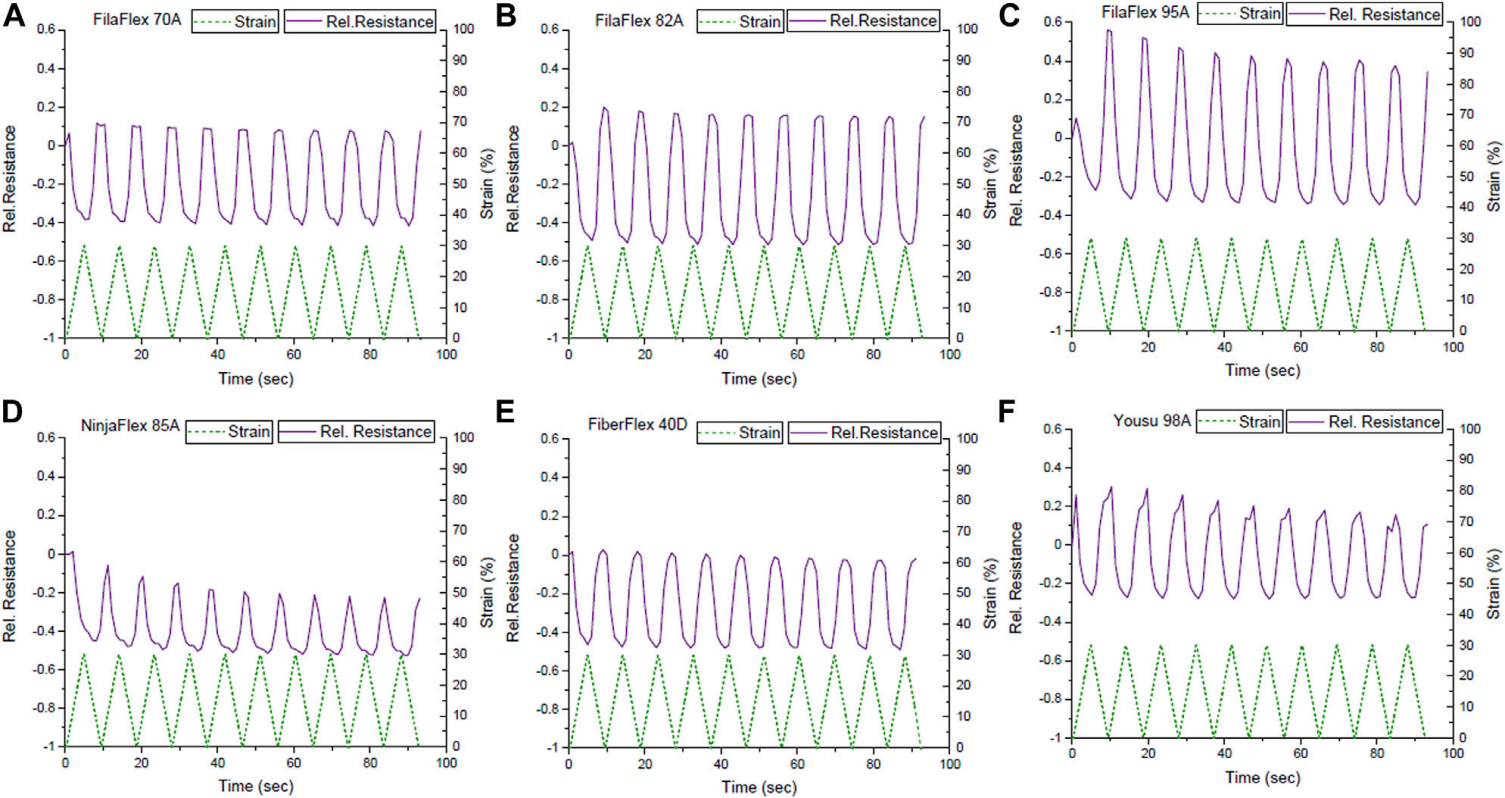
Sensor response during dynamic tensile testing between 0 and 30% strains for the different TPU strips with *in situ* integrated strain sensing elements; **(A)** FilaFlex 70A, **(B)** FilaFlex 82A, **(C)** FilaFlex 95A, **(D)** NinjaFlex 85A, **(E)** FiberFlex 40D, and **(F)** Yousu 98A.

Figure 4 presents the dynamic testing between 0 and 30% strains for the different TPU combinations produced using the multi-material FDM printer. Looking at the sensor signal, during the dynamic testing, it can be seen that the relative resistance behavior did not follow the applied strain (Figure 4). As expected from Figure 3B, at the beginning of the first cycle, a small increase in the relative resistance can be observed. Afterward, the relative resistance decreased while increasing the strain up to 30% strain. After the first cycle, the relative resistance decreased when the strain increased and *vice versa*. Additionally, at the area of strains where the buckling occurred and the mechanical stress became zero or even negative (Supplementary Figure S2B), a plateau for the electrical signal can be observed, too. This plateau caused an uncertainty in the sensor response (Supplementary Figure S2A). However, with the exception of the strips made with FilaFlex 70A and Yousu 98A filaments, the uncertainty is minimal and almost negligible for all other TPU strips (Table 2). For the sensitivity of the sensor response, expressed by the change in the relative resistance, it can be seen that there is a trend in getting a higher sensitivity with the increasing Shore hardness for the three FilaFlex samples. For the drift of the sensor signal between the different cycles, no significant difference can be observed between the different strips, except the TPU strips printed with the NinjaFlex 85A filament. As for the hysteresis in the electrical signal, it can be seen (Table 2) that there is no trend between the values of the hysteresis and the Shore hardness of the TPU. However, the hysteresis was higher in the cases where the uncertainty was also large (FilaFlex 70A and Yousu 98A), showing a link between the two values.

**FIGURE 5:**
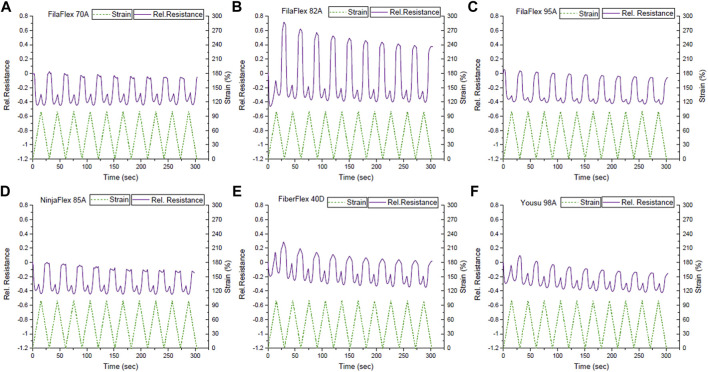
Sensor response during dynamic tensile test between the strains 0 and 100% for the TPU strips with integrated strain sensing elements from **(A)** FilaFlex 70A, **(B)** FilaFlex 82A, **(C)** FilaFlex 95A, **(D)** NinjaFlex 85A, **(E)** FiberFlex 40D, and **(F)** Yousu 98 A.

Based on the relative resistance–strain curve up to the point of fracture (see Figures 3A,B), in addition to the dynamic test between 0 and 30% strains, the dynamic tensile testing was also performed, between 0 and 100% strains (Figure 5).

As expected from Figure 3B, in the first cycle, the resistance slightly increased, followed by a decrease and an increase when the strain increased from 0 to 100%. However, a plateau in the sensor signal can be observed.

The uncertainty appeared at higher strains than what was observed in the range of strains 0–30% (Table 3). Buckling was also present in the range of strains 0–100% (Supplementary Figure S5), but it appeared at higher strains than the buckling in the range of 0–30%. Comparing the sensor response during the dynamic testing at the two different ranges of strain, there can be found a correlation with the slope of the curve of the relative resistance with strain during the tensile test up to the point of fracture (Figure 3). At the range of strains 0–30%, the slope of the curve was negative, and in the dynamic test, the sensor response was reversed, but the linearity was good, with the exception of the plateau caused by the buckling (Supplementary Figure S4). In the case of the 0–100% range of strain, the slope changes from negative to positive, and as a consequence, there is a secondary peak and insufficient linearity. In this range of strains, a higher signal drift was observed in the strips printed with the FilaFlex 82A filament and a lower drift for the strips printed with the FilaFlex 95A filament (Table 3).

**TABLE 3:** Strain values where buckling and uncertainty can be overserved as well as the signal drift at 100% strain. All data have been calculated for the dynamic tensile tests at strain 0–100%. The buckling is derived from Supplementary Figure S5. The uncertainty and drift are derived from Figure 5.

Substrate material	Buckling at strain (%)	Uncertainty at strain (%)	Signal drift (%) at strain 100%
FilaFlex 70A	23	69	7
FilaFlex 82A	25	76	53
NinjaFlex 85A	24	88	6
FiberFlex 40D (∼90A)	37	80	7
FilaFlex 95A	33	72	6
Yousu 98A	32	71	63

**FIGURE 6:**
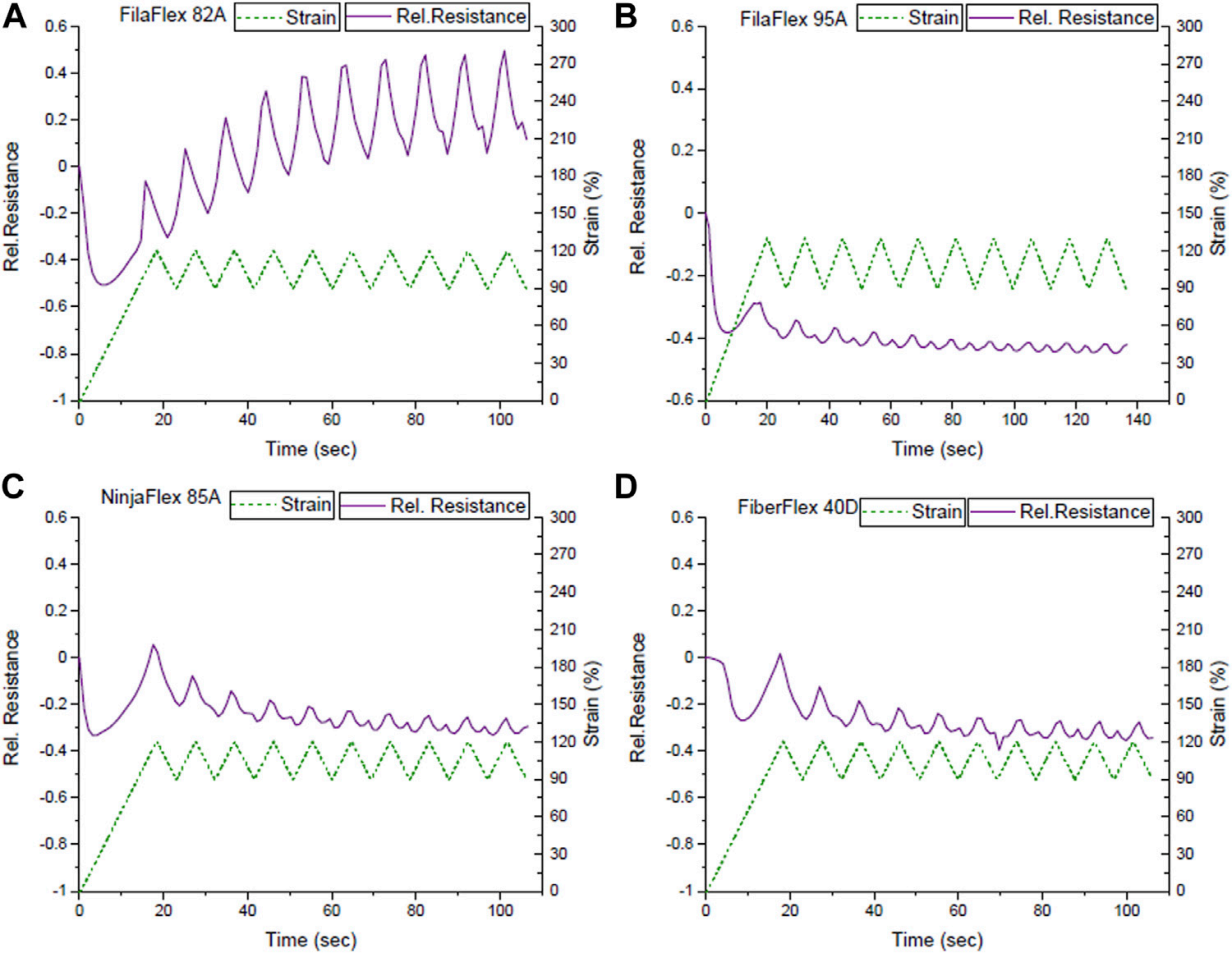
Sensor response during dynamic tensile test between the strains 90 and 120% for the TPU strips with integrated strain sensing elements from **(A)** FilaFlex 82A, **(B)** FilaFlex 95A, **(C)** NinjaFlex 85A, **(D)** FiberFlex 40D.

To avoid the change of the electrical resistance slope from negative to positive during the dynamic cycling tests, the TPU strips were pre-strained and cycled between 90 and 120% strains (Figure 6). Based on Figure 3B, it was expected that the relative resistance increases while increasing the strain. Therefore, a monotone increase and decrease in the sensor signal was expected for straining and releasing, respectively.

From the mechanical response of the sensors (Supplementary Figure S6), it was seen that the effect of the buckling in the unloading for the mechanical stress could be avoided (Table 4). Unfortunately, the TPU strips made with FilaFlex 70A and Yousu 98A filaments broke during cycling. For the electrical response, it was seen that the signal drift increased significantly at high strains. As expected, for the first cycle, a monotonic increase and decrease in the electrical sensor signal was seen. However, after a few cycles, a secondary peak appeared during the unloading, and therefore, the uncertainty in the signal response appeared again (Figure 6). With the exception of the FilaFlex 82A, in all the other TPU strips, the uncertainty appeared at high strain (Table 4).

**TABLE 4:** Strain values where buckling and uncertainty were overserved as well as the signal drift at 120% strain for the TPU strips with integrated sensing elements. All data have been calculated for the dynamic tensile tests at strain 90–120%.

Substrate material	Uncertainty at strain (%)	Signal drift (%)
FilaFlex 82A	94	145
NinjaFlex 85A	104	107
FiberFlex 40D (∼90A)	120	19
FilaFlex 95A	108	70

### Stress and Sensor Signal Relaxation

An important aspect of the sensors used in robotic applications, such as robotic grippers, is the fact that the gripper moves at position open or position close and maintains that position over some time. For that reason, a test of the mechanical and electrical relaxation could provide very useful information for the potential use of the sensor elements in soft robotic gripper systems. Because of the previous dynamic tensile test results, the test was performed at the range of strains between 0 and 30%, where the sensors responded linearly during the dynamic tensile test. In this test, the goal was to quantify the stress relaxation and the relaxation of the electrical signal, which was calculated at static strain 30% (Table 5).

**TABLE 5:** Mechanical and electrical relaxation for TPU strips with the integrated *in situ* sensing elements, measured during the dynamic tensile test with a dwell time of 30 s at 30% strain during the third cycle. The mechanical relaxation is derived from Supplementary Figure S7 and the electrical relaxation from Figure 7.

Substrate material	Mechanical relaxation (%)	Electrical relaxation (%)
FilaFlex 70A	12.4	11.2
FilaFlex 82A	14.2	8.9
NinjaFlex 85A	16.6	8.5
FiberFlex 40D	10.6	5.5
FilaFlex 95A	17.0	8.4
Yousu 98A	29.3	4.2

**FIGURE 7:**
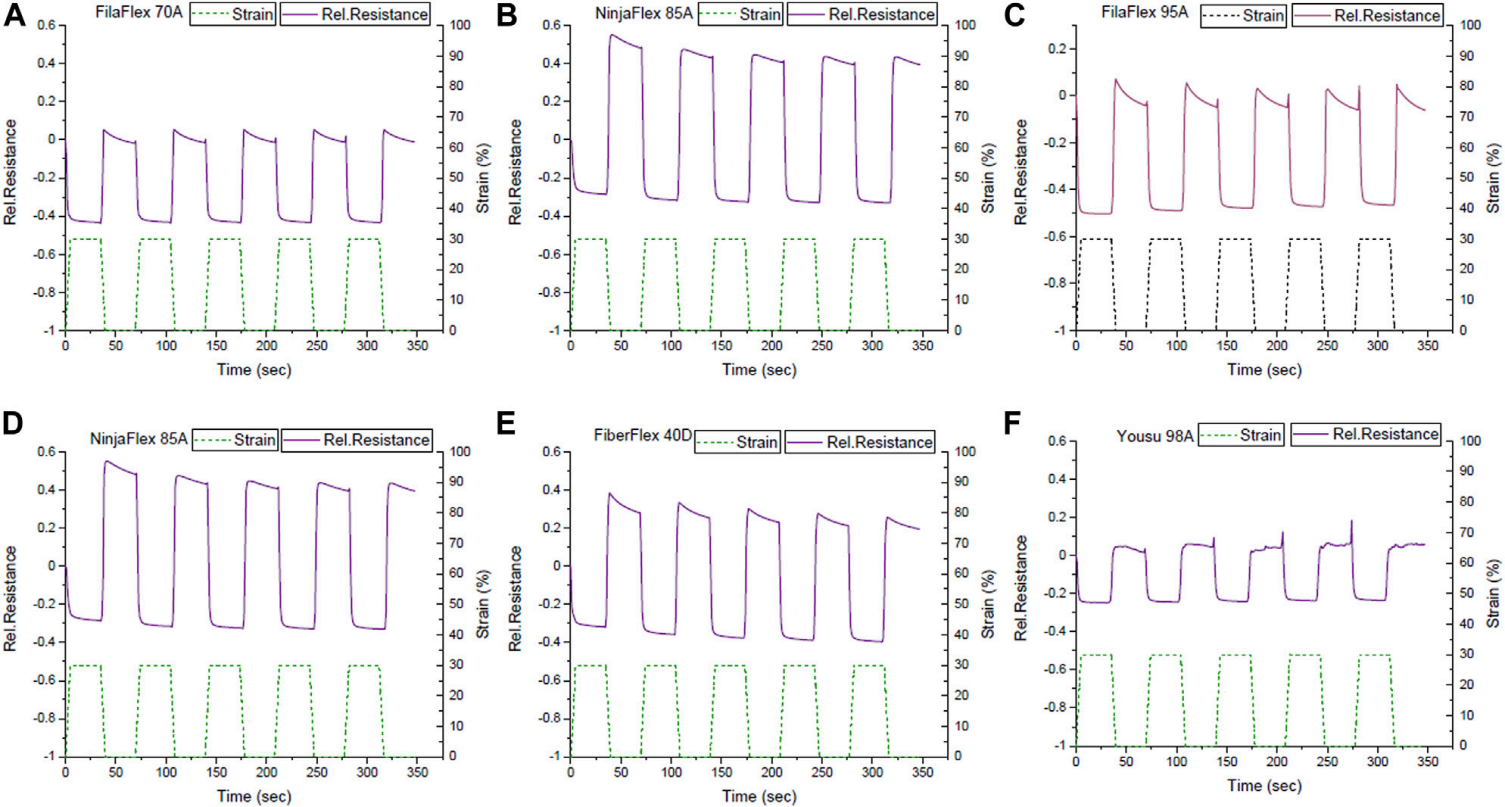
Signal response during the dynamic tensile test between the strains 0 and 30% with a dwell time of 30 s at 0% and 30 s at 30% strain for the TPU strips with integrated *in situ* sensing elements with a substrate; **(A)** FilaFlex 70A, **(B)** FilaFlex 82A, **(C)** FilaFlex 95A, **(D)** NinjaFlex 85A, **(E)** FiberFlex 40D, and **(F)** Yousu 98A.

From measuring the electrical signal relaxation, it was seen that the systems responded linearly without the appearance of a secondary peak (Figure 7). This is in good agreement with the dynamic tensile test results (Figure 5).

For the TPU strip with the lowest Shore hardness, the relaxation of the signal was higher than for all the other strips. With an increase in the Shore hardness, the relaxation of the signal decreased. For the TPU strips made of FilaFlex 82A, NinjaFlex 85A, and FilaFlex 95A filaments, this trend was observed. The TPU strips based on the Yousu 98A filament had the highest Shore hardness and the lowest signal relaxation of all the strips. However, for the strips fabricated with the Yousu 98A filament, there was significant signal noise observed, especially at low strains. This noise was not seen at the other systems, and it is not obvious where this noise in the electrical signal comes from. The strips made with the Yousu 98A and FiberFlex 40D filaments had lower relaxation than all others.

The stress relaxation increased by increasing Shore hardness, except for the FiberFlex 40D (Table 5). The FiberFlex 40D had the lowest stress relaxation of all the strips (Supplementary Figure S5). The systems with substrate FilaFlex 82A and FilaFlex 85A combine low drift and relatively low relaxation. Especially the case of FiberFlex 40D combines a stress relaxation of only 10.6%, a signal relaxation of 5.5%, and signal drift lower than those of the systems of higher Shore hardness than 85A.

### Application: Gripper With Integrated Sensing Elements

A soft TPU-based gripper with integrated sensing elements was printed with multi-material FDM with the purpose of demonstrating the practical use of the *in situ* printed piezoresistive sensing elements (Figure 2). Therefore, the Eel filament was used for printing the sensing element, and the other TPU filaments were used to print the body of the soft gripper. Tendons attached to a servomotor were used for the motion of the soft gripper. A maximum deformation of 30% on the bottom side of the band, where the sensor element was printed, was calculated. Because of the high uncertainty for TPU strips based on FilaFlex 70A and the Yousu 98A filaments mentioned in Table 2, soft gripper structures were not produced with these two materials.

Looking at the values of the initial resistance (Table 6), it can be seen that the value of the initial resistance increased with the Shore hardness of the TPU-based soft gripper structure. A similar trend was seen in the case of the TPU strips with integrated sensing elements before (see Table 2).

**TABLE 6:** Values of the initial electrical resistance of the soft gripper with *in situ* printed sensing elements.

Substrate material	Shore hardness after printing (A)	R_o_ (kΩ)	Coefficient of variation (%)
FilaFlex 82A	78	17 ± 5	29
NinjaFlex 85A	81	28 ± 6	21
FiberFlex 40D	87	39 ± 7	18
FilaFlex 95A	91	72 ± 3	4

**FIGURE 8:**
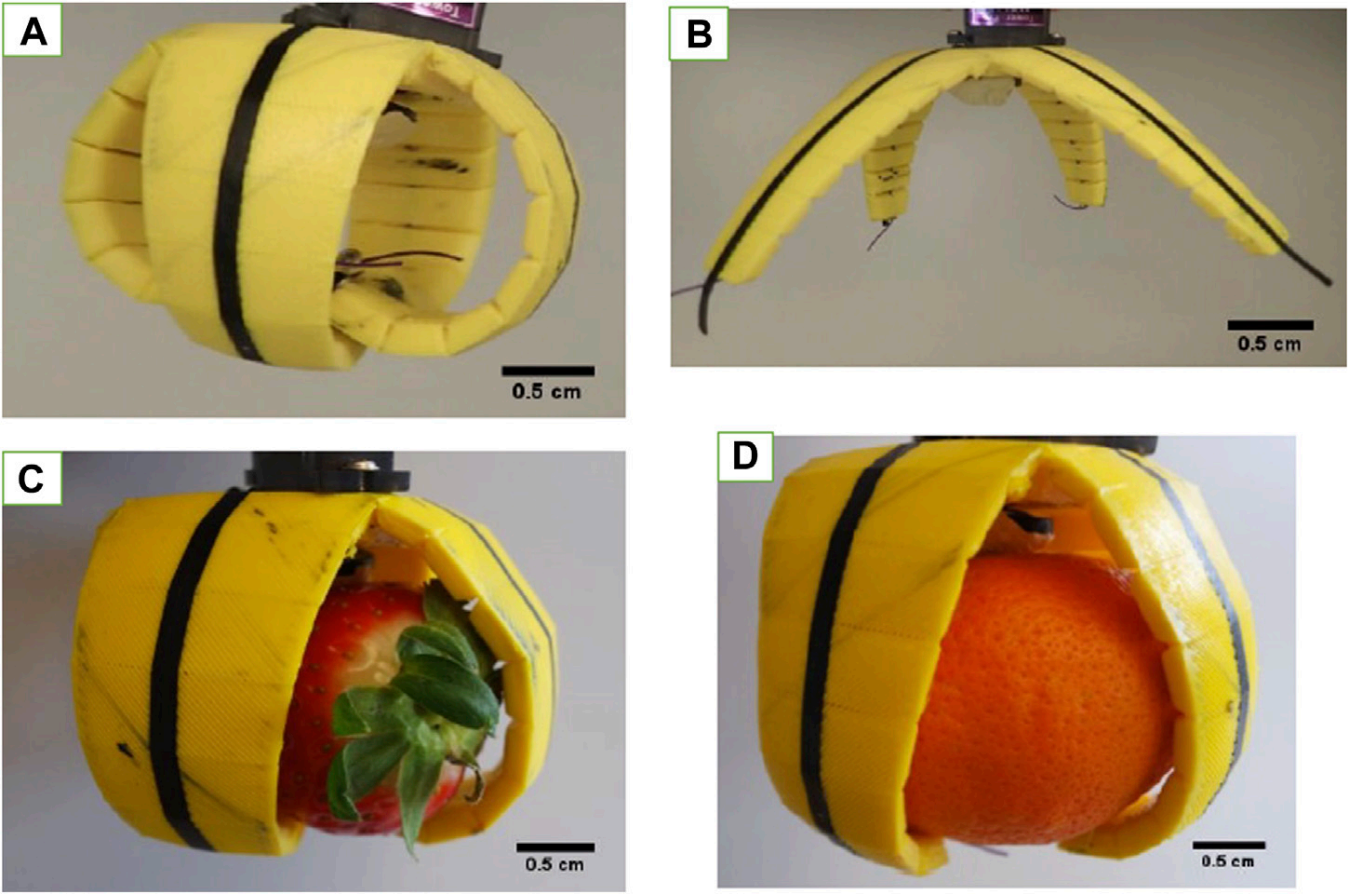
Gripper with the integrated sensing elements in position **(A)** closed, **(B)** open, **(C)** gripping a small object, and **(D)** gripping a big object.

A test with five cycles of opening and closing the tentacles of the soft gripper was performed, to evaluate the electrical resistive behavior of the *in situ* printed sensor elements. The test was performed to distinguish between the open and closed positions and between gripping small and big fruit objects (Figure 8).

The initial resistance changed in a similar way, as is seen in Table 2 with the TPU strips. Strips made with lower–Shore hardness filaments have a lower resistance than those made out of filament with higher Shore hardness. Even though the printing parameters and the geometrical structure were equal for all used TPU filament materials, a difference in the sensor response was observed. For the soft gripper made with the FilaFlex 82A filament (the lowest Shore hardness), the resistance and the change in resistance were the lowest. For the soft gripper structure produced with the NinjaFlex 85A filament, a secondary peak was seen when the soft gripper moved from the open to the closed position. For better comparison, the relative resistance change, between the open and closed positions, was calculated for all soft grippers. For the FilaFlex 82A–, NinjaFlex 85A–, FiberFlex 40D–, and FilaFlex 95A–based soft grippers, a relative resistance change of 9, 24, 29, and 46% was observed, respectively. Therefore, the change in relative resistance, which is similar to the sensitivity of the sensor response, is increasing when the TPU filament with higher Shore hardness was used. Both the differences in the resistance and the relative resistance seem to originate from the different Shore hardnesses of the substrate. A similar observation was described by Georgopoulou et al. (2020a), where the Shore hardness of the silicone matrix affected the sensor properties of an SEBS-based sensor fiber. Additionally, a test including the gripping of a small and a big object was performed for the soft grippers made with the different TPU filaments. In the case of the smaller object, the deformation of the soft gripper caused a larger change in the electrical signal of the integrated sensor element than in the case of the bigger object.

**FIGURE 9:**
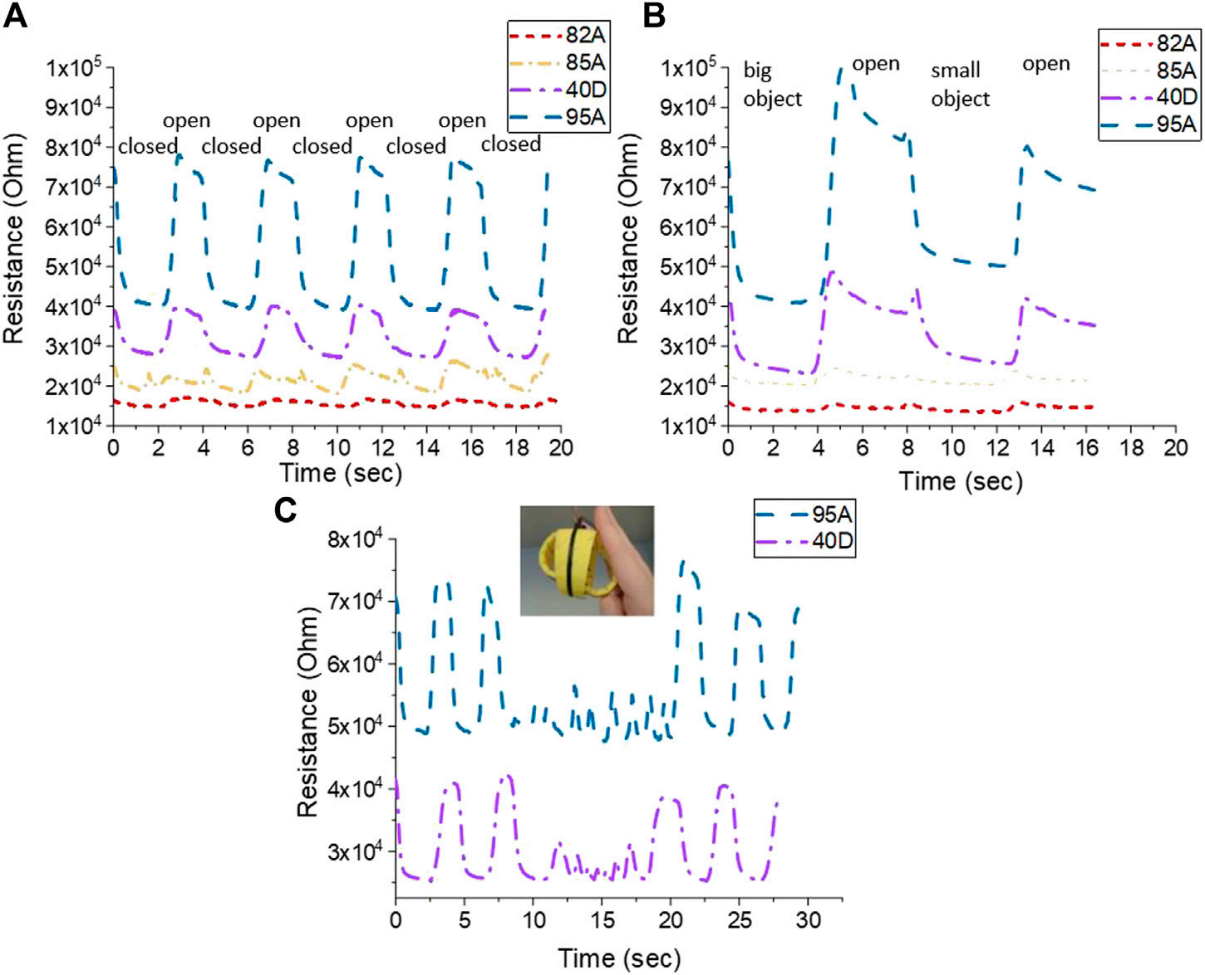
Electrical signal from the *in situ* printed sensing element on the soft robot gripper structures, using different TPU filaments, when **(A)** cycling between the open and closed positions, **(B)** gripping a big and a small object, and **(C)** during moving between the open and close positions with and without an obstacle present. The obstacle was imposed at the time point 10 s and lasted for about 10 s.

Similar to previous results, for the two soft grippers made with FilaFlex 82A and NinjaFlex 85A filaments, it was not possible to distinguish between the big and small objects using the electrical signal of the integrated sensor. For the soft grippers based on the FiberFlex 40D filament and the FilaFlex 95A filament, it was possible to detect a signal difference of the sensor element for the big and small objects (Figure 9B). For the soft grippers made with the FiberFlex 40D and FilaFlex 95A filaments, an additional test of obstacle recognition was performed. In this test, the gripper was programmed to operate by moving between the open and closed positions, but after two cycles, an obstacle was positioned to prevent the gripper from opening. As shown in Figure 9C, the change in the resistance before, during, and after the obstacle can be observed.

During the first two opening and closing cycles, the resistance changes as expected. During the third cycle, the soft gripper is blocked during the opening movement. A sudden drop in the electrical resistance sensor value at the point where the obstacle was imposed could be observed. After the removal of the obstacle, the resistance of the sensor element recovered to the initial value of the open position and the opening and closing cycle of the soft gripper followed again. Similar to the previous test, the best performance was achieved using the soft gripper structure produced with the filament with the highest Shore hardness.

## Discussion

In this study, the challenge of integrating functional elements like sensors in soft robotics was addressed using AM. Multi-material FDM was used for printing the sensors and the body of the gripper using a one-step process. The *in situ* integration of the sensors has significant advantages in efficiency in production. No additional steps, like curing post-printing, were required in this case of using FDM. Since different filaments based on TPU exist in the market, a goal in this study was to investigate which one of these materials would be the best choice as a substrate for the sensor integration to result in the optimal sensor performance. For the printing, parameters had to be optimized for the case of using flexible filaments. Optimizing parameters like using lower printing speed and large extrusion multiplier compared to what is reported in the literature for other thermoplastic materials was necessary for 3D printing with the flexible elastomers. In comparison to standard thermoplastics for FDM printing, lower printing speed and higher extrusion multiplier were needed to achieve a good quality of the 3D-printed TPU structures. The defining property that was different for all filaments was the Shore hardness of the TPU filaments. The dimensions of the substrates were different from what was expected after the printing process. The length was not affected, but the width and thickness were different for the different substrates. Even though a clear trend was not visible, it was seen that the substrates of higher Shore hardness had a larger width and thickness than the substrates of low Shore hardness, and this fact was attributed to a higher die swell effect for the higher–Shore hardness TPU filaments. Different steps were taken in the characterization process of the sensor, starting from a tensile test up to the point of fracture and continuing with tensile testing under dynamic and quasi-static conditions.

The first investigation was the tensile test up to the point of fracture, and as expected, it was seen that the stiffness of the sensor increased with the Shore hardness of the substrate. A correlation between the Shore hardness of the substrate and the Young’s modulus showed an exponential relationship between the two parameters. It is common practice in the field of elastomer materials to use the Shore hardness instead of the Young’s modulus to describe the stiffness of an elastomer. The correlation between the two sizes shows that the practice of using the Shore hardness instead of the Young’s modulus is well founded, especially at low Shore hardness, because of the exponential relationship.

As for the sensor response during the same test, it was seen that the slope of the relative resistance with the strain changed from positive to negative and then to positive again. The profile piezoresistive behavior of thermoplastic elastomers with conductive fillers has been already investigated by others. Flandin et al. (2001) described the four different regimes of piezoresistive behavior when straining up to the point of fracture. The first area with the resistance increasing was described as the initiation phase, characterized by the breakage of the conductive network. At the second area, the resistance decreases, and this area was described as the reversible area in which rotation of the electrical filler occurs, which results in a denser conductive network after reaching the lowest. At the third area, the conductive network was preserved, and this was called the area of recoverable damage, and finally, the forth area, called the area of depercolation, was characterized by the breakdown of the conductive network. Similar behavior has been reported for SEBS-based piezoresistive sensors by Mattmann et al. (2008) and Culha et al. (2014). Looking on the TPU strips printed with the three different filaments above, we can see the same four regimes, discussed by Flandin et al. and other authors. Because of this effect, the strain levels were varied for the later dynamic cycling measurements to detect the optimal strain level area for future applications. Looking at the response of the different TPU strips with different Shore hardnesses, no clear trend could be seen at this point between sensor sensitivity and the Shore hardness of the TPU strip. A trend could be seen between the initial resistance and the Shore hardness of the substrate, as selecting a substrate of lower Shore hardness resulted in lower resistance value. A similar trend was seen in the cross-section of the sensing element on the strips. The cross-section decreased with an increasing Shore hardness. Such differences can be associated with the printing process and the die swell effect that increased with the increasing Shore hardness. When the printing head is printing on a substrate with a lower Shore hardness, the distance of the nozzle to the substrate is larger, resulting in the cross-section of the printed sensing element being rounder with a larger area. A flatter cross-section with a smaller area can be achieved by printing on a substrate with a higher Shore hardness. These differences can explain the trend in the *R*_*0*_ values, but they cannot directly explain the differences in the relative resistance.

Additionally, a substrate of lower Shore hardness resulted in better consistency in the quality of the samples, a parameter linked directly with the printing process. Because the printing parameters were the same for all the filaments with the different Shore hardnesses, it is possible to attribute the differences and the trend seen in the electrical behavior to the composition of the different filaments. The materials used in this attempt are commercially available filaments, but nonetheless, it is known that in order to alter the Shore hardness in thermoplastic elastomers, the composition and especially the ratio between hard and soft segments of the thermoplastic elastomer are often altered (Puszka, 2018). Such alteration could potentially have an impact on the interface and adhesion of the sensing element on the substrate, leading to the trend in the behavior of the electrical signal.

Durng the tensile test under dynamic conditions, the first range of strain applied was 0–30%, and at this range, the negative piezoresistive response was observed. Negative piezoresistive behavior has already been reported for polydimethylsiloxane-based strain sensors (Chowdhury et al., 2019), and it has been attributed to the viscous effect of the viscoelastic behavior of the thermoplastic elastomer matrix. The change of the sensor behavior after the first cycle has already been reported by a previous work on 3D-printed single Eel filaments (Georgopoulou et al., 2020c). Based on those two references, we propose that the different sensing behavior between the first and the second cycle is caused by the viscous part of the viscoelastic behavior of TPU. This is a well-known phenomenon for TPU, and it is otherwise known as creep (Yang et al., 2015). The entanglement of the elastomer chains, during the unloading, will result in a hindering of the reconstruction of the carbon filler network. Comparing the different TPU strips, it can be seen that the uncertainty in the sensor signal was different for the different cases. The high value of the uncertainty for the FilaFlex 70A and Yousu 98A meant that these TPU strips were quite unsuitable to be used in this range of strains.

The same test was repeated for the range of strains 0–100%. This range includes the first, second, and third areas as described by Flandin et al. (2001). In this range of strains, a plateau in the sensor signal was observed. This plateau fits very well with the plateau in the stress–strain measurements and can be therefore explained by the viscous behavior of the TPU materials used for this study. It is worthwhile to mention that such a sensor behavior cannot be used to monitor the strain inside a soft robotic structure because the same rel. resistance will result in different strain levels. In comparison to the dynamic tensile test before (0–30%), buckling and uncertainty were observed at significantly high strain values. This behavior implies that the viscous part of the viscoelastic behavior of the printed TPU dominated the mechanical and resistance behavior and a higher remaining deformation occurs.

The same test was then repeated at strains 90–120%, and at this range, even if the buckling disappeared, the uncertainty did not disappear. A secondary peak appeared during the unloading phase of the cycle. This uncertainty in combination with the large drift meant that this range of strains was also not appropriate to get a good sensor response. The presence of a secondary peak in the electrical signal for both the ranges 0–100% and 90–120%, in combination with the significant drift of the relative resistance, implies that commercial TPU materials are not recommended for strain sensor applications at higher strain levels (e.g., above 30% strain).

As for the response of the signal and the mechanical stress, during the quasi-static testing, there was the presence of relaxation in both cases. No trend could be found in the values of the relaxation with the Shore hardness of the TPU strip. However, even though a trend was not seen, based on a combination of small relaxation and drift, some TPU strips seemed to have the best characteristics of the sensor behavior (FiberFlex 40D, followed by FilaFlex 82A and FilaFlex). For the sensor strips made with the FilaFlex 70A, FilaFlex 95A, FiberFlex 40D, and Yousu 98A filaments, the appearance of a spike at the sensor signal at the end of the dwell time at 0% strain could be observed. This spike appeared when the strain started to increase and was not considered a part of the relaxation but rather an artifact of the measurement. This artifact can be associated with the initiation region or region I as described in a previous study (Flandin et al., 2001). They claim that the increase in strain causes a rearrangement in the conductive network and results in a spike observed at the beginning of the loading. Their explanation fits well in this case, since the artifact is observed only at the beginning of the straining but not at the unloading phase of the test. Overall, the tensile testing of the strips helped to distinguish some of the characteristics that could guide the material selection. These observations were linked with the sensitivity, consistency in quality, and the selection of the appropriate range for the sensor function. This type of testing can help reduce the number of prototypes fabricated for testing the different combinations in the future and guide the material selection from the material characterization step already. This can help eliminate some candidate material combinations, and as a result, fewer prototypes need to be fabricated.

As for the testing performed with the soft robotic grippers with the integrating sensing elements, the TPU material used for the gripper was selected from the cases in sensor characterization that showed small uncertainty. Even though the linearity was good in the sensor response, the signal at positions open and closed, where there was a dwell time present, showed significant relaxation. This fact implies that this system with the chosen sensor cannot be used for precise monitoring of the position of the gripper but rather to show if the gripper is in position open or closed. Nonetheless, because of the sensitivity of the sensor, it is possible to distinguish, based on the value of the resistance in position closed, if the gripper is gripping a small or a large object. Based on this characteristic, despite the limitation that derives from the relaxation of the electrical signal, the sensor attributes some functions to the gripper like obstacle recognition and object size identification. A common application for these soft grippers is the careful handling of sensitive objects like foodstuff. A suggested application for the sensorized gripper used in this study is packaging of fruit. Based on the size of the fruit, the signal of the sensor could indicate what type of fruit is being gripped, and the packaging would be coordinated accordingly. Moreover, the signal of the sensor indicated when a blockage prevented the gripper from opening. This could be a useful characteristic in the performance of the gripper, as the sensor signal could alert the user when interference would be needed to remove the blockage.

As for the material selection, from the tests with the different grippers, it was seen that using a TPU of higher Shore hardness had significant advantages compared with the TPU of lower Shore hardness. In the systems with higher Shore hardness, the sensitivity of the integrated sensing element was higher than that in the other systems. This effect was also seen in the dynamic test 0–30% for the three strips based on FilaFlex. It is well known that for soft robotic systems, low Shore hardness is preferential. However, using design aspects like thinner walls and low infill during 3D printing will result in a reduced stiffness of the soft gripper structure. This observation can be a useful guide for material selection when designing soft robots with integrated sensing elements. Even though these commercial TPU materials have some limitations in the limiting range of their function (0–30%), by choosing the appropriate mechanical characteristics of the TPU (Shore hardness), a good sensor response can be obtained especially for distinguishing when the gripper is in position open or closed.

## Conclusion

Multi-material FDM can be used for fabricating structures with integrated sensors for soft robotic applications. In this attempt, piezoresistive sensors were *in situ* integrated onto a 3D-printed TPU structure. A commercial conductive filament, consisting of thermoplastic polyurethane (TPU) and carbon black (CB), was used as a sensor material. Strips and soft gripper structures made by TPU filaments with different Shore hardnesses were combined. The analysis of the piezoresistive signal of the integrated sensor elements was investigated by dynamic and quasi-static tensile tests. The results showed that the TPU printed strip structures could be used up to 30% strain before the viscous part of the viscoelastic behavior of the elastomer dominated the piezoresistive signal behavior. At higher strains, a loss of linearity and large uncertainty due to the dominant viscous behavior of the TPU materials could be observed. The increase of the viscous behavior also affected the drift of the piezoresistive signal negatively.

In this study, it could be demonstrated that the relaxation of the electrical signal is affected by the Shore hardness of the used TPU filaments. The electrical signal relaxation decreased with increasing the Shore hardness. Unfortunately, the drift of the electrical signal increased with increasing the Shore hardness of used TPU filaments. We assume that a combination of printed TPU structures with piezoresistive TPU-based sensing elements is not promising for future soft robotic applications with large elongation (deformations). However, the tensile testing helped indicate characteristics of the sensor performance for the different substrate/sensor combinations, like the appropriate range of application, consistency in sample quality, and sensitivity of the sensor. Such analysis in the future can help reduce the number of prototypes and tests that need to be performed to select the appropriate material.

The performance of the integrated sensing elements was also tested by using an open-source soft robotic gripper. In this case, it was seen that using a material with higher Shore hardness led to better sensitivity. In comparison to soft grippers made with lower Shore hardness TPU filaments, the difference in resistance between the opening and closing positions was significantly larger. The integrated sensor elements on soft robotic grippers made with high–Shore hardness TPU filaments showed the ability to distinguish between the positions open and close and big and small objects, and finally, they could indicate when an obstacle was preventing the gripper from opening. It can be assumed that commercially available TPU conductive and nonconductive filaments are not perfect for the piezoresistive sensor integrated soft robotic structure. However, a limited range of strains can be used for monitoring the motion of soft robotic grippers. In a case demonstrated in the study (sensor printed on a FiberFlex 40D) substrate, there could be seen a good compromise between sensitive sensor response and stiffness of the substrate. This case can be an example for guiding the selection of material combinations for soft robotic systems produced with FDM.

## Data Availability

The raw data supporting the conclusions of this article will be made available by the authors, without undue reservation.

## Supplementary Material

Supplementary Table S1 Measurements of the length, width, and thickness of the substrates of the strips after printing. The measurements were derived from three different samples.

Supplementary Figure S1 TPU strip with an integrated *in situ* strain sensor printed using a multi-material FDM printer. The TPU substrate (orange) and the strain sensing element (black) printed with the Eel filament.

Supplementary Figure S2 Calculation of the **(A)** uncertainty, **(B)** buckling, and **(C)** signal drift during the dynamic tensile testing.

Supplementary Figure S3 Cross-section of the samples, measured using the optical microscope, for the samples **(A)** FilaFlex 70A, **(B)** FilaFlex 82A, **(C)** NinjaFlex 85A, **(D)** FiberFlex 40D, **(E)** FilaFlex 95A, and **(F)** Yousu 98A.

Supplementary Figure S4 Dynamic tensile testing between the strains 0% and 30% of 3D-printed TPU strips with *in situ* integrated strain sensing elements; **(A)** FilaFlex 70A, **(B)** FilaFlex 82A, **(C)** FilaFlex 95A, **(D)** NinjaFlex 85A, **(E)** FiberFlex 40D, and **(F)** Yousu 98A.

Supplementary Figure S5 Dynamic tensile test between the strains 0% and 100% for TPU strips with integrated strain sensing elements from **(A)** FilaFlex 70A, **(B)** FilaFlex 82A, **(C)** FilaFlex 95A, **(D)** NinjaFlex 85A, **(E)** FiberFlex 40D, and **(F)** Yousu 98A.

Supplementary Figure S6 Dynamic tensile test between the strains 90% and 120% for the TPU strips with integrated strain sensing elements from **(A)** FilaFlex 82A, **(B)** FilaFlex 95A, **(C)** NinjaFlex 85A, and **(D)** FiberFlex 40D.

Supplementary Figure S7 Stress response during the dynamic tensile test between the strains 0% and 30% with a dwell time of 30 seconds at 0% and 30 seconds at 30% strain for the TPU strips with *in situ* integrated sensing elements with a substrate; **(A)** FilaFlex 70A, **(B)** FilaFlex 82A, **(C)** FilaFlex 95A, **(D)** NinjaFlex 85A, **(E)** FiberFlex 40D, and **(F)** Yousu 98A.
