# Supplementary figures and images for: Fabrication of a Soft Robotic Gripper With Integrated Strain Sensing Elements Using Multi-Material Additive Manufacturing
Source: Front Robot AI. 2021 Nov 1;8:615991. doi: 10.3389/frobt.2021.615991 (PMC8965514; doi:10.3389/frobt.2021.615991)

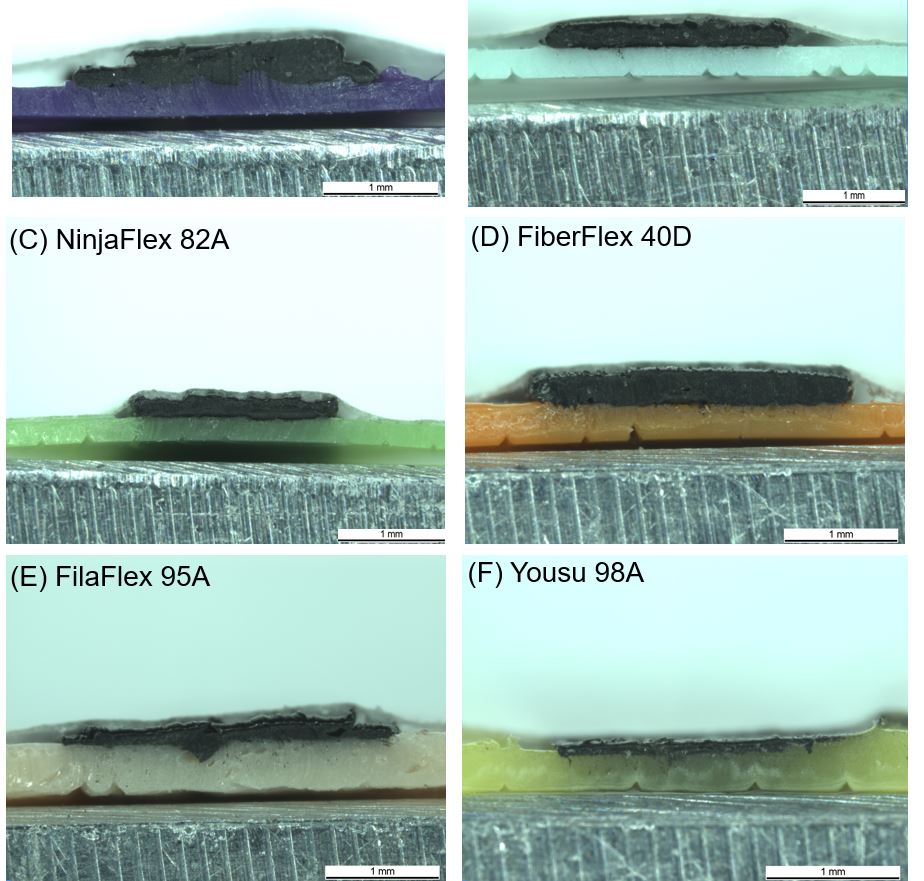

Supplement: Supplementary file 1 [file Image3.jpg]

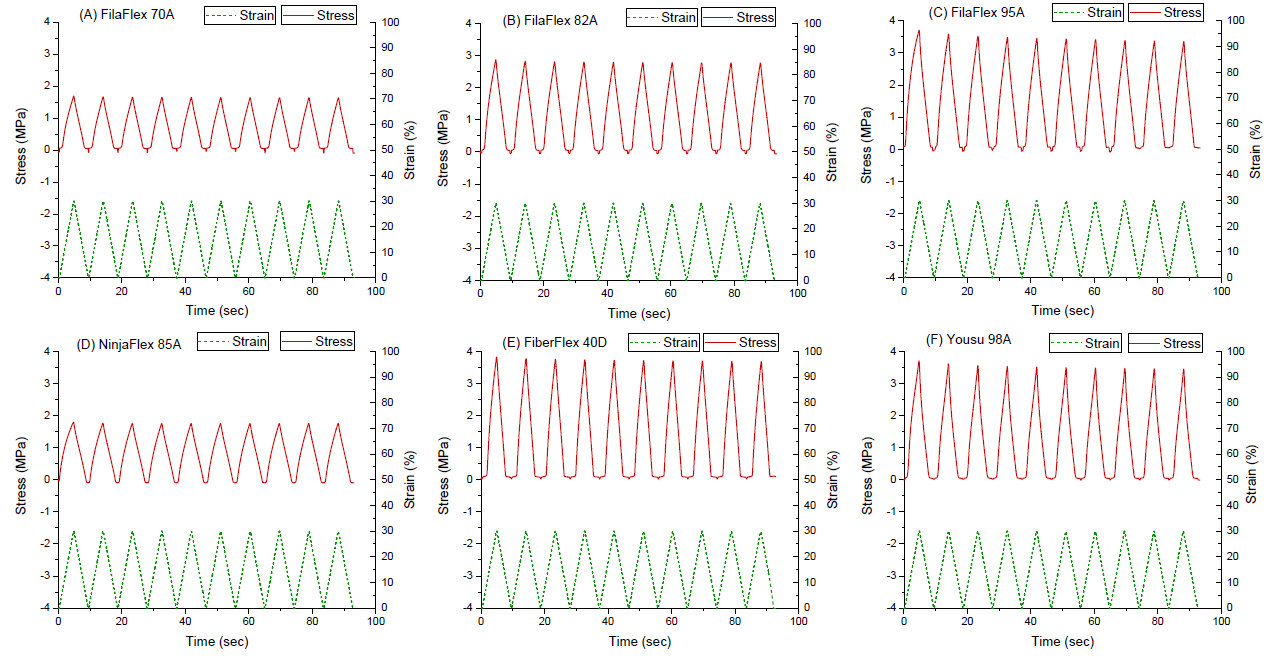

Supplement: Supplementary file 2 [file Image6.TIF]

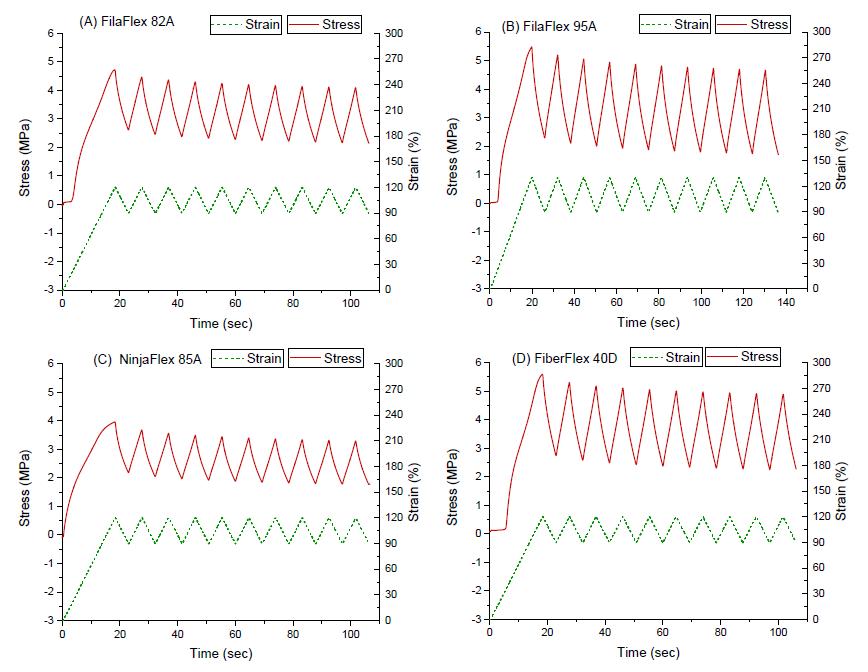

Supplement: Supplementary file 4 [file Image4.TIF]

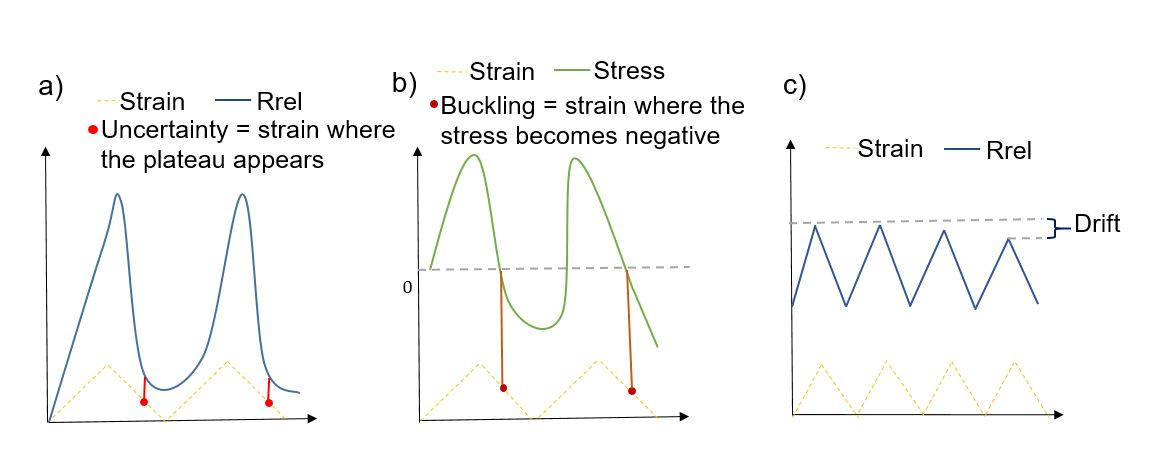

Supplement: Supplementary file 5 [file Image2.JPEG]

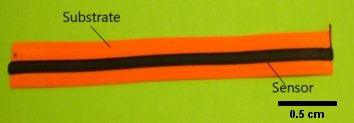

Supplement: Supplementary file 6 [file Image1.TIF]

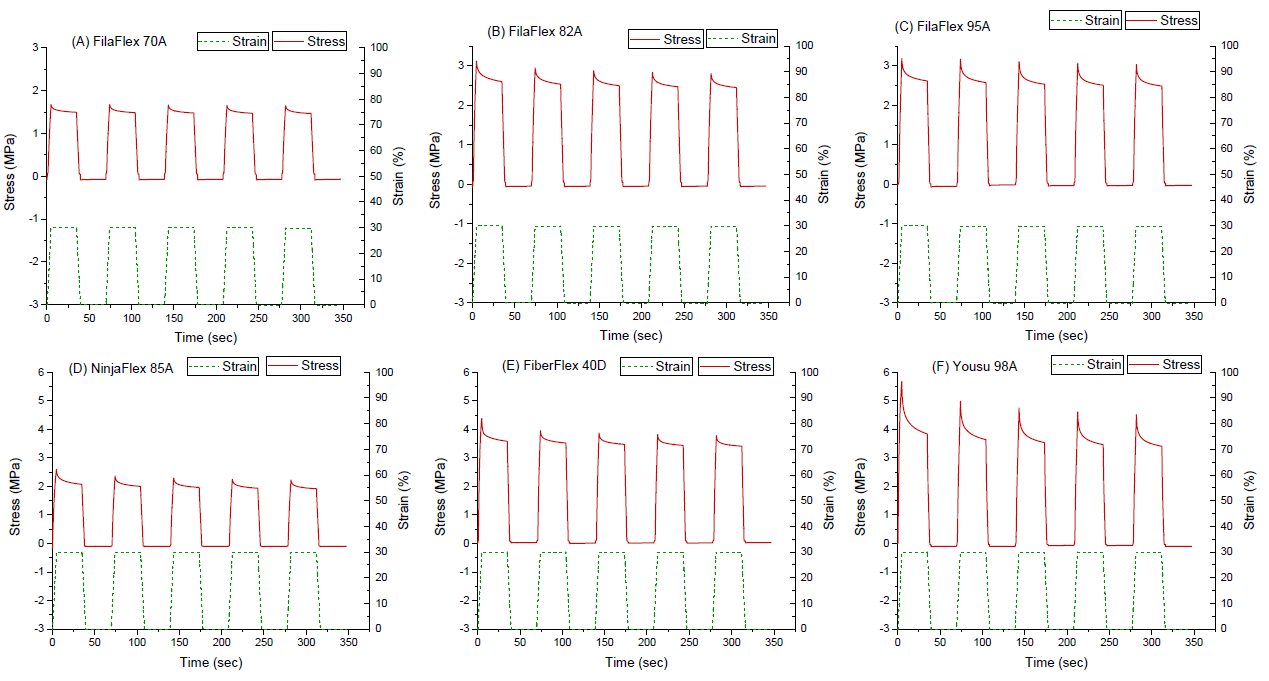

Supplement: Supplementary file 7 [file Image7.tif]
