# Supplementary material for: Fabrication of a Soft Robotic Gripper With Integrated Strain Sensing Elements Using Multi-Material Additive Manufacturing
Source: Front Robot AI. 2021 Nov 1;8:615991. doi: 10.3389/frobt.2021.615991 (PMC8965514; doi:10.3389/frobt.2021.615991)
Supplement: Supplementary file 3 [file Table1.DOCX]

| Substrate | Length (mm) | Width (mm) | Thickness (mm) |
| --- | --- | --- | --- |
| FilaFlex 70A | 130 ± 2 | 10.48 ± 0.09 | 0.34 ± 0.02 |
| FilaFlex 82A | 130 ± 1 | 10.13 ± 0.05 | 0.31 ± 0.01 |
| NinjaFlex 85A | 130 ± 2 | 10.13 ± 0.007 | 0.32 ± 0.02 |
| FiberFlex 40D (~92A) | 130 ± 1 | 10.23 ± 0.03 | 0.38 ± 0.02 |
| FilaFlex 95A | 130 ± 4 | 10.62 ± 0.16 | 0.47 ± 0.05 |
| Yousu 98A | 130 ± 2 | 10.61 ± 0.17 | 0.54 ± 0.03 |
