# Supplementary material for: Fabrication of a Soft Robotic Gripper With Integrated Strain Sensing Elements Using Multi-Material Additive Manufacturing
Source: Front Robot AI. 2021 Nov 1;8:615991. doi: 10.3389/frobt.2021.615991 (PMC8965514; doi:10.3389/frobt.2021.615991)
Supplement: Supplementary file 8 [file DataSheet1.pdf]

Metadata note

Image 1 is referred in the text as Supplementary Figure 1.

Image 2 is referred in the text as Supplementary Figure 2.

Image 3 is referred in the text as Supplementary Figure 3.

Image 4 is referred in the text as Supplementary Figure 4.

Image 5 is referred in the text as Supplementary Figure 5.

Image 6 is referred in the text as Supplementary Figure 6.

Image 7 is referred in the text as Supplementary Figure 7.

Table 1 is referred in the text as Supplementary Table 1.
